# Efficacy-specific herbal group detection from traditional Chinese medicine prescriptions via hierarchical attentive neural network model

**DOI:** 10.1186/s12911-021-01411-2

**Published:** 2021-02-18

**Authors:** Li Chen, Xinglong Liu, Siyuan Zhang, Hong Yi, Yongmei Lu, Pan Yao

**Affiliations:** 1 Department of Computer Science, Sichuan University, Chengdu, China; 2 School of Basic Medicine, Chengdu University of TCM, Chengdu, China

**Keywords:** Efficacy-specific herbal group, Hierarchical attentive neural network, Traditional Chinese medicine, Prescription

## Abstract

**Background:**

Mining massive prescriptions in Traditional Chinese Medicine (TCM) accumulated in the lengthy period of several thousand years to discover essential herbal groups for distinct efficacies is of significance for TCM modernization, thus starting to draw attentions recently. However, most existing methods for the task treat herbs with different surface forms orthogonally and determine efficacy-specific herbal groups based on the raw frequencies an herbal group occur in a collection of prescriptions. Such methods entirely overlook the fact that prescriptions in TCM are formed empirically by different people at different historical stages, and thus full of herbs with different surface forms expressing the same material, or even noisy and redundant herbs.

**Methods:**

We propose a two-stage approach for efficacy-specific herbal group detection from prescriptions in TCM. For the first stage we devise a hierarchical attentive neural network model to capture essential herbs in a prescription for its efficacy, where herbs are encoded with dense real-valued vectors learned automatically to identify their differences on the semantical level. For the second stage, frequent patterns are mined to discover essential herbal groups for an efficacy from distilled prescriptions obtained in the first stage.

**Results:**

We verify the effectiveness of our proposed approach from two aspects, the first one is the ability of the hierarchical attentive neural network model to distill a prescription, and the second one is the accuracy in discovering efficacy-specific herbal groups.

**Conclusion:**

The experimental results demonstrate that the hierarchical attentive neural network model is capable to capture herbs in a prescription essential to its efficacy, and the distilled prescriptions significantly could improve the performance of efficacy-specific herbal group detection.

## Background

Traditional Chinese Medicine (TCM) has a long history of several thousand years, and during such long period massive prescriptions are accumulated. These prescriptions were providing effective protection for ancestors of Chinese people from different diseases for thousands of years, and are applied widely even nowadays [1]. However, the prescriptions are formed by different people at different historical times empirically or with trial and error, without agreed and rigorous theoretical support or specification, which results in an uneven quality in these prescriptions and further hinders them from more extended applications in modern society. In order to refine these massive prescriptions and discover valuable patterns of herbal usages in TCM behind the prescriptions, data mining and machine learning technologies are incorporated in recent years [2, 3] such as association rule mining to discover herbal groups for alopecia treatment [4] and for syndrome differentiation of TCM [5], random walk and label transmission to detect herbal groups [6], multi-content Latent dirichlet allocation(LDA) to recommend prescriptions for the patients of amenorrhea and lung cancer [7], and supervised learning to perform classification for TCM clinical records [8]. Among all the issues tried with data mining and machine learning technologies, detecting essential herbal groups for a particular efficacy is crucial for the science of TCM formulas as well as for the practical functionality of the TCM prescriptions in modern society [6, 9]

The efficacy of a prescription is not simply the addition of all herbs. On the contrary, they interact with each other and show better curative efficacy and fewer side effects than a single one. Besides, the efficacy of a single herb generally is diverse, and practitioners need to control single herbs with multi-efficacy to play an expected efficacy during diagnosing disease and making prescriptions. For example, the efficacies of *Rhizoma Rhei*(  ) are *heat-clearing and detoxicating*, *removing accumulation with purgation*, and so on. When combined with *Coptis chinensis*(  ) and *Radix Scutellariae*(  ), *Rhizoma Rhei* can deliver the efficacy of *heat-clearing and detoxicating*. On the other hand, *Rhizoma Rhei* plays the efficacy of *removing accumulation with purgation* when combined with *mirabilite* (  ). Hence, TCM doctors not only know the efficacy of herbs but also master the efficacy of herbal groups when making prescriptions.

Therefore, TCM researchers draw more and more attentions recently in herbal group mining, and gain some promising results [10–13]. However, most existing methods for the task determine essential herbal groups based only on the raw frequencies with which herbal groups occur in a collection of prescriptions. Although other variations to these raw-frequency-based methods exist, such as the one using random walk technique on a herb graph based on a similarity measurement between herbs with their features [6, 14], in fact they are all heuristic and feature-engineering-based, treating herbs with different surface forms orthogonally and overlooking entirely the fact that prescriptions in TCM are formed empirically by different people at different historical times, and thus full of herbs with different surface forms expressing the same material, or even noisy and redundant herbs.

In order to overcome the difficulties mentioned above, in this paper we devise a two-stage approach to detect efficacy-specific herbal groups (ESHGs) from prescriptions in TCM. The first stage is performing recognition of essential herbs in a prescription for its efficacy, and the second stage collects all prescriptions of an efficacy with their respective essential herbs to discover ESHGs. For the first stage a hierarchical attentive neural network model is employed, and by means of its attention mechanism, essential herbs in a prescription for its efficacy are recognized. For the second stage frequent pattern mining is incorporated to discover the ESHGs of an efficacy from its distilled prescriptions obtained in the first stage. We conduct a series of experiments to verify the effectiveness of our two-stage approach from two angles. The first one is the ability of the attention mechanism to capture essential herbs for a prescription efficacy and thus distill the prescription for subsequent frequent-pattern-based ESHGs detection, and the second one is the quality of the ESHGs discovered with our two-stage approach. The experimental results demonstrate that the hierarchical attentive neural network model is capable to capture herbs in a prescription essential to its efficacy, and the distilled prescriptions significantly improve the effect of ESHGs detection.

The major contributions of this work are three-fold: (1) a deep learning model is incorporated into the ESHGs detection task, where a dense real embedding is learned for every herb to capture semantical correlations among herbs, thus overcoming enormous differences in herbal name usages in the long period of TCM history; (2) a hierarchical attentive mechanism is proposed to capture essential herbs in a prescription for its efficacy to distill the prescription for the purpose of improving ESHGs detection; (3) a series of experiments is conducted to investigate the performance of our model for identifying herbs in a prescription essential to its efficacy and detecting ESHGs for a particular efficacy, and the experimental results verify the effectiveness of our two-stage approach.

**Fig. 1:**
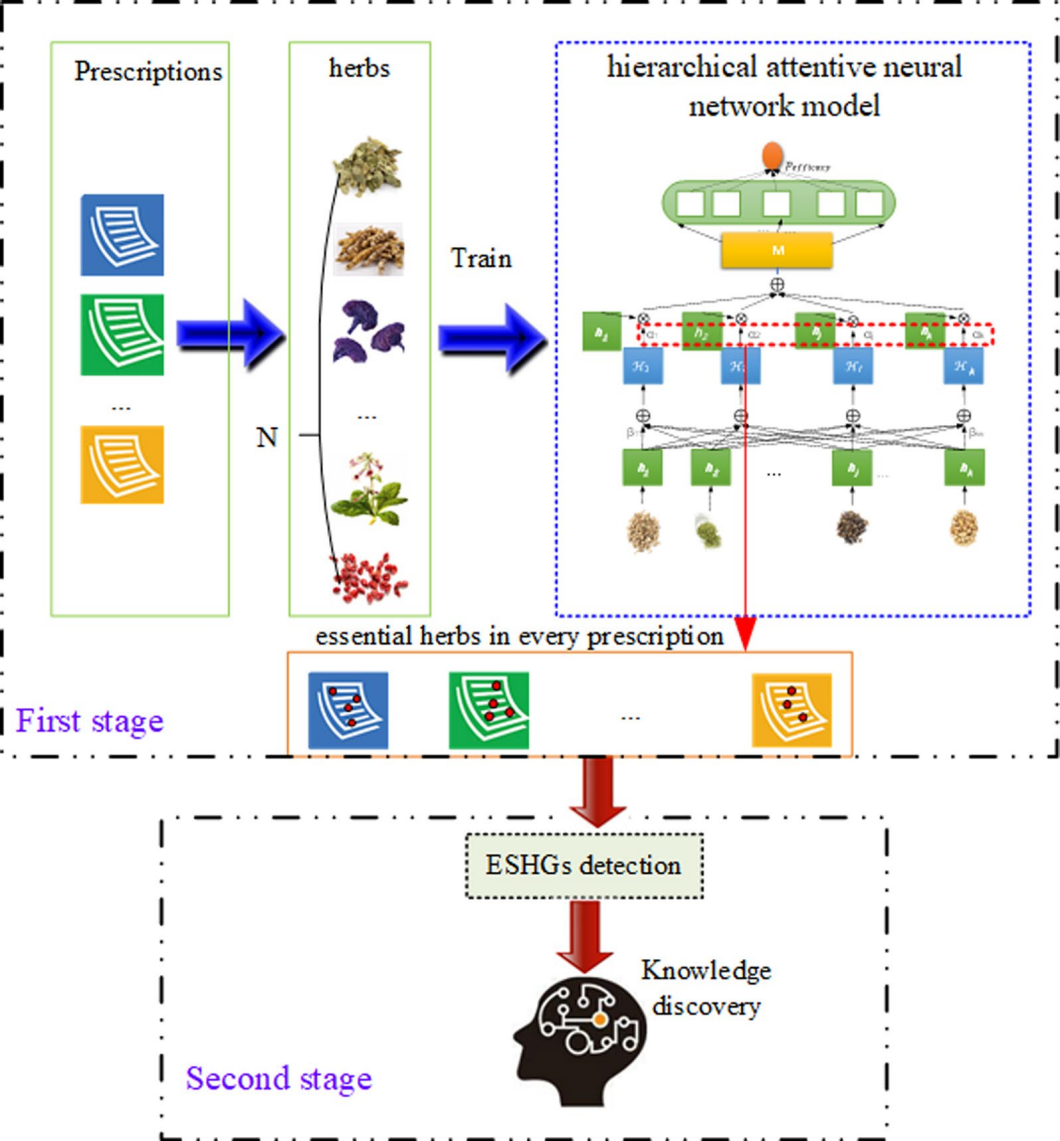
The two-stage approach for ESGHs detection

**Fig. 2:**
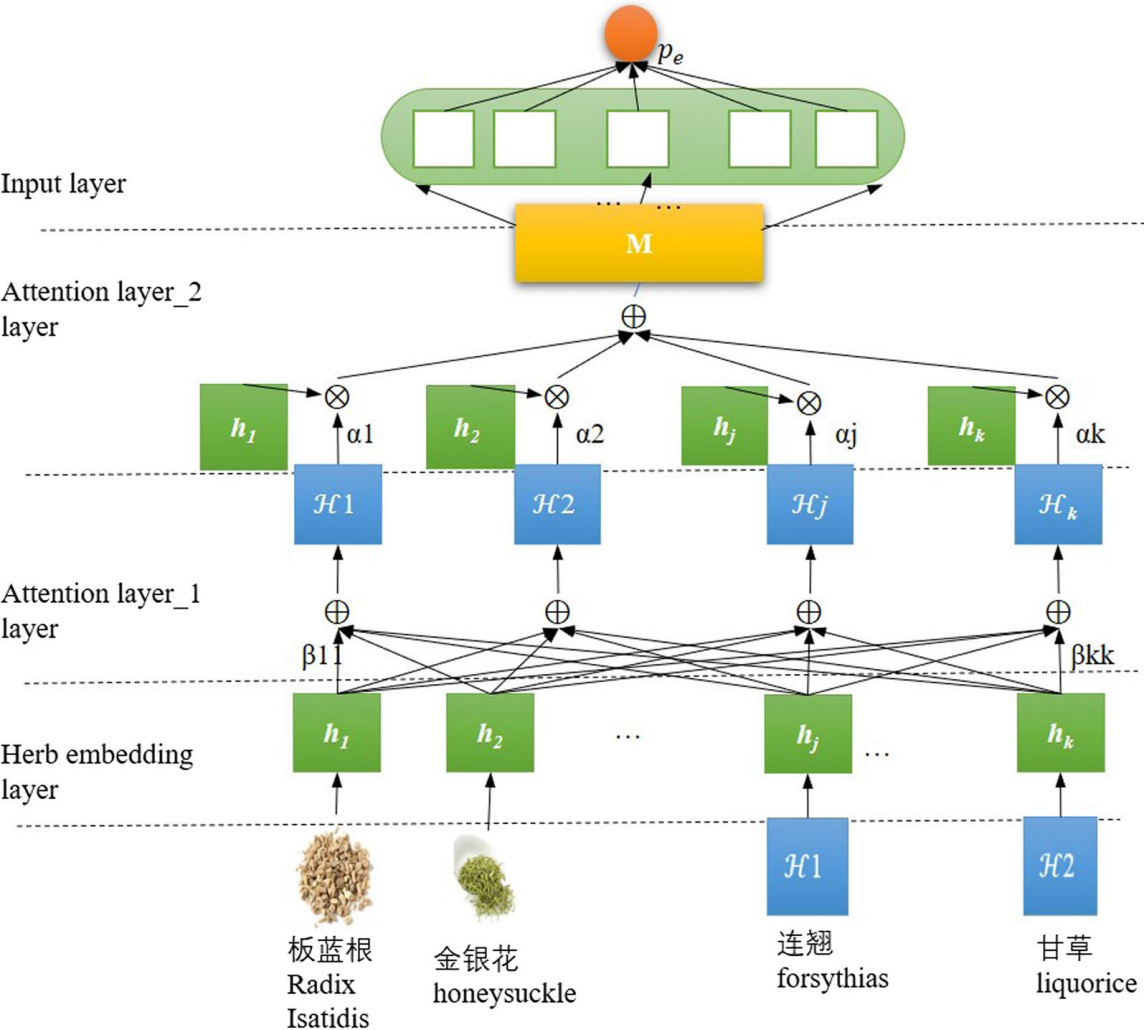
The architecture of the hierarchical attentive neural network

**Fig. 3:**
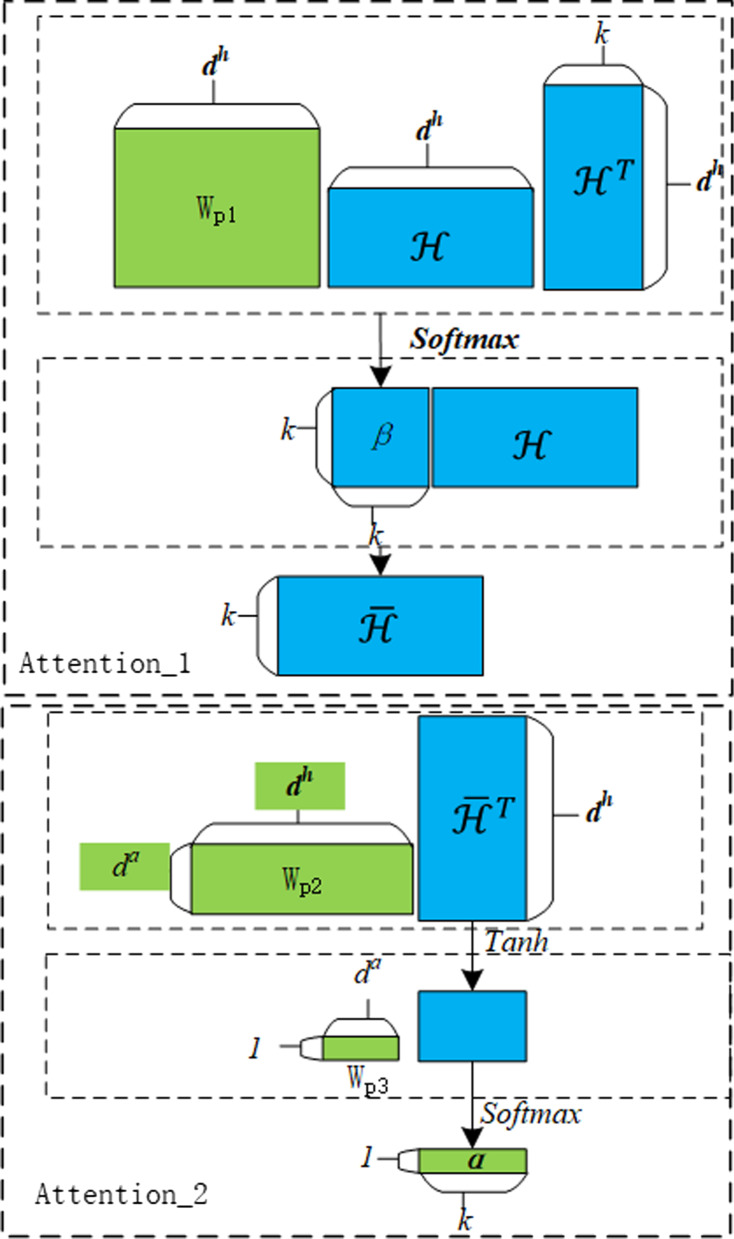
The hierarchical attentive structure

**Fig. 4:**
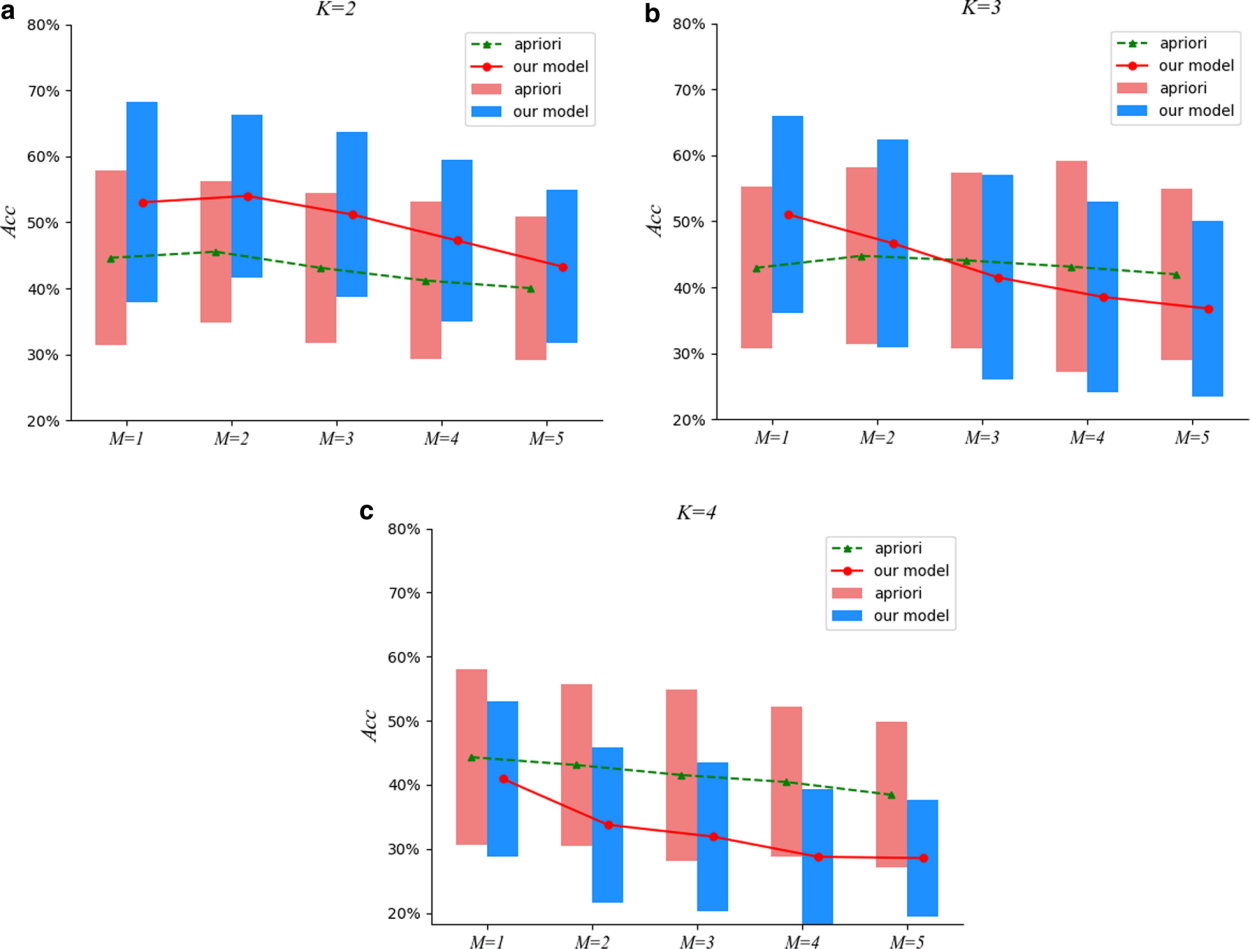
The comparison results under $M=5$

**Table 1:**
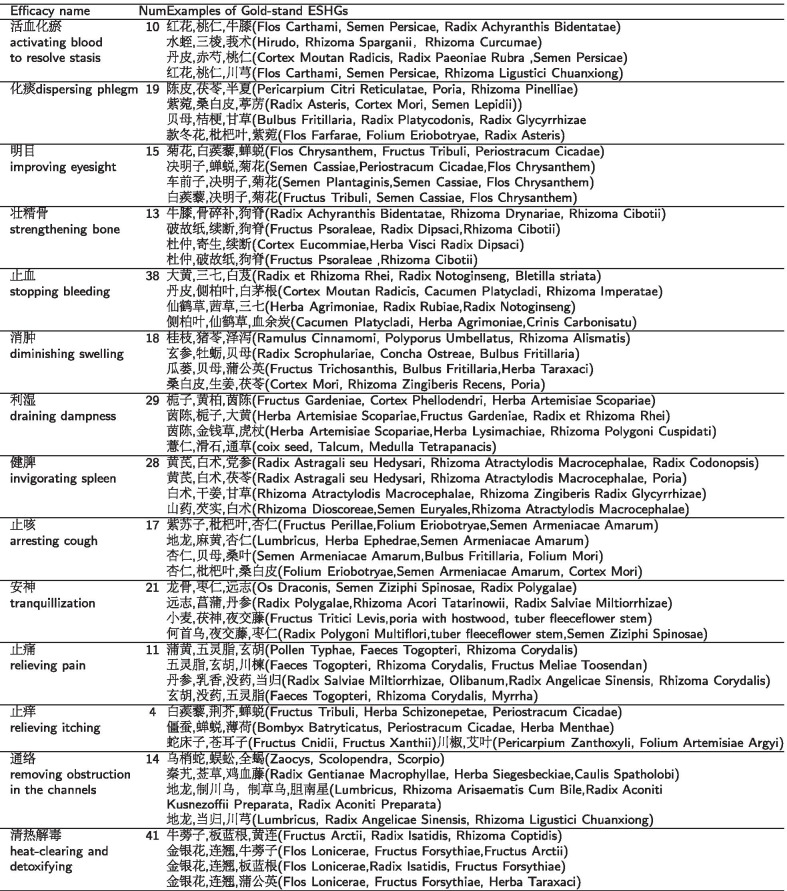
The numbers of ESHGs and examples for each efficacy

Displayed is the number of instances, for each MTU group and all combined MTU, where the MTU quality evaluation metric was either (+) improved, (−) worse, or (±) no change. For each MTU group, sample size was 48 (8 MTU × 6 subjects). For all combined MTU, sample size was 144 (8 per group × 3 groups × 6 subjects). Statistically significant differences are denoted using coloured cells, where green indicates improvement, red indicates worsening, and yellow indicates no change. In cases of no statistically significant difference between any of the three types of results, cells were left unshaded

+, Represents improvement from models 1–3; −, represents worsening from models 1–3; **±** represents no change between models 1 and 3

**Table 2:**
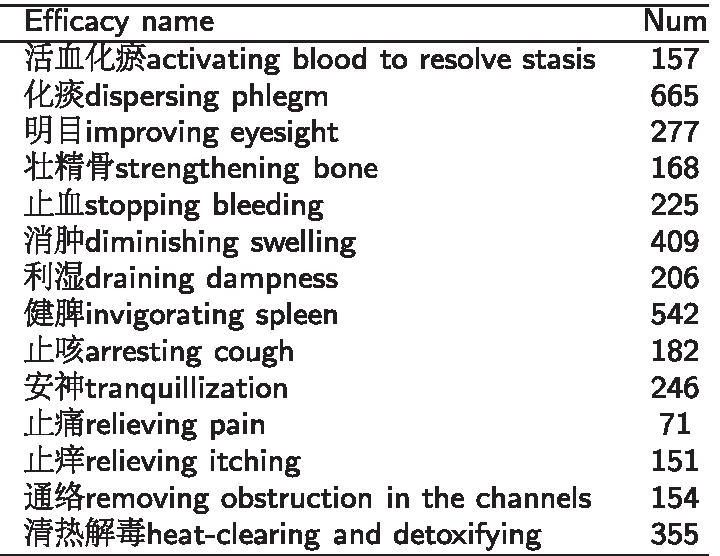
The numbers of prescriptions for each efficacy

Displayed is the number of instances, for each MTU group and all combined MTU, where the MTU quality evaluation metric was either (+) improved, (−) worse, or (±) no change. For each MTU group, sample size was 48 (8 MTU × 6 subjects). For all combined MTU, sample size was 144 (8 per group × 3 groups × 6 subjects). Statistically significant differences are denoted using coloured cells, where green indicates improvement, red indicates worsening, and yellow indicates no change. In cases of no statistically significant difference between any of the three types of results, cells were left unshaded

+, Represents improvement from models 1–3; −, represents worsening from models 1–3; **±** represents no change between models 1 and 3

## Related work

In recent years, many disciplines witness rapid growth in employing data-intensive approaches based on machine learning and data mining technologies to discover patterns hidden in a massive volume of data. Likewise, some efforts have emerged in TCM recently which utilize machine learning and data mining technologies for discovering knowledge from TCM literature [15–18], clinical records [8, 19, 20], and prescriptions [12, 13, 21–26]. Among all these data mining tasks the last one draws particular attention due to the fact that, prescriptions as the primary knowledge sources for TCM are invented mostly with empirical experiences in a long historical span of times and distilling knowledge from them are far from completion, which hinders applications of TCM in modern society. Data mining and machine learning from prescriptions are hopeful to cover theoretical gaps in TCM and lift practical performance in health care and disease treatment.

With regard to types of knowledge to be discovered from prescriptions in TCM, herbal groups having a particular efficacy are of great significance for TCM theory as well as for its practice, thus becoming a primary focus in recent works on TCM prescription data mining and knowledge discovery. The efficacy of herb has been identified and summarized through serval thousand years of clinical practices, but there are many herbal groups for disease/symptom/efficacy still needed to be further mined. Current approaches to perform such task proposed by now are mostly utilizing co-occurrence frequency of herbs in a prescription collection to measure their strengths in forming a herbal group with a particular efficacy or toward a particular disease/symptom. While initially the Apriori algorithm or its some accelerated variations are employed in these frequency-based approaches [10–13, 18, 27] due to their simplicity and ease of implementation, recently Han et al. [13] proposed to mine frequent patterns not only in occurrence of herbs, but also in their absence in a collection of prescriptions. The purpose of the latter is to discover simultaneously collocated as well as contraindicated combinations of herbs for a particular disease, thus potentially having more applications. However, the above proposed approaches, including the one in [13], are all lacking in empirical evaluation for their quality of the generated herbal groups, and the only evaluation reported in their original papers are efficiency in terms of the time expended for the mining. Furthermore, the raw-frequency-based approaches treat different herbs equally, ignoring skew distribution in their usage frequencies and their special properties, which obviously should result in poor quality herbal groups.

In order to overcome the difficulties faced by raw-frequency-based approaches, Wang et al. [6] incorporated features of herbs into the mining process, such as their nature and flavor. They measure the similarity for a pair of herbs according to their features and then construct a weighted undirected graph with the vertexes denoting herbs and the edges weighted with the corresponding similarity, and then a random walk processing is performed on the graph to discover required herbal groups. Yao et al. [21] proposed an LDA-based topic model to capture latent syndromes and herbal roles (i.e. *jun-chen-zuo-shi*) in a prescription, thus a group of herbs for a set of symptoms can be inferred by the model.

Although the recent studies such [6] and [21] have made significant progress in TCM prescription mining, almost all of them treat the herbs orthogonally, representing them with one-hot manner. Such manner has to be faced with the difficulties of data sparsity. Furthermore, the one-hot manner in essence ignores possible correlations on the level of the representation, which is necessary for TCM herbs. Prescriptions of TCM are formed by different people at different historical times empirically in a way of trial and error, without agreed and rigorous theoretical support or specification. The forming process has resulted directly in an uneven quality in the prescriptions being full of herbs with different surface forms expressing the same material, and also the same surface forms denoting distinct herbs. Without distilling such formed prescriptions, the raw-frequency-based and one-hot-based approaches are difficult to achieve satisfying prescription mining results.

To tackle the issues mentioned above, a two-stage approach to detect essential groups for a particular efficacy from TCM prescriptions is proposed in this paper. In the first stage, we devise a neural network model to distill prescriptions in TCM, in which herbs are embedded into dense real vectors learned automatically to capture the semantical correlations, and a hierarchical attentive mechanism is proposed to recognize essential herbs in a prescription for its efficacy, filtering out insignificant herbs. For the second stage, ESHGs are mined from the distilled prescriptions acquired in the first stage. Finally, we collect a set of efficacies labeled with their known herbal groups by TCM experts as the gold-standard test data to verify the effectiveness of our approach in discovering ESHGs.

**Fig. 5:**
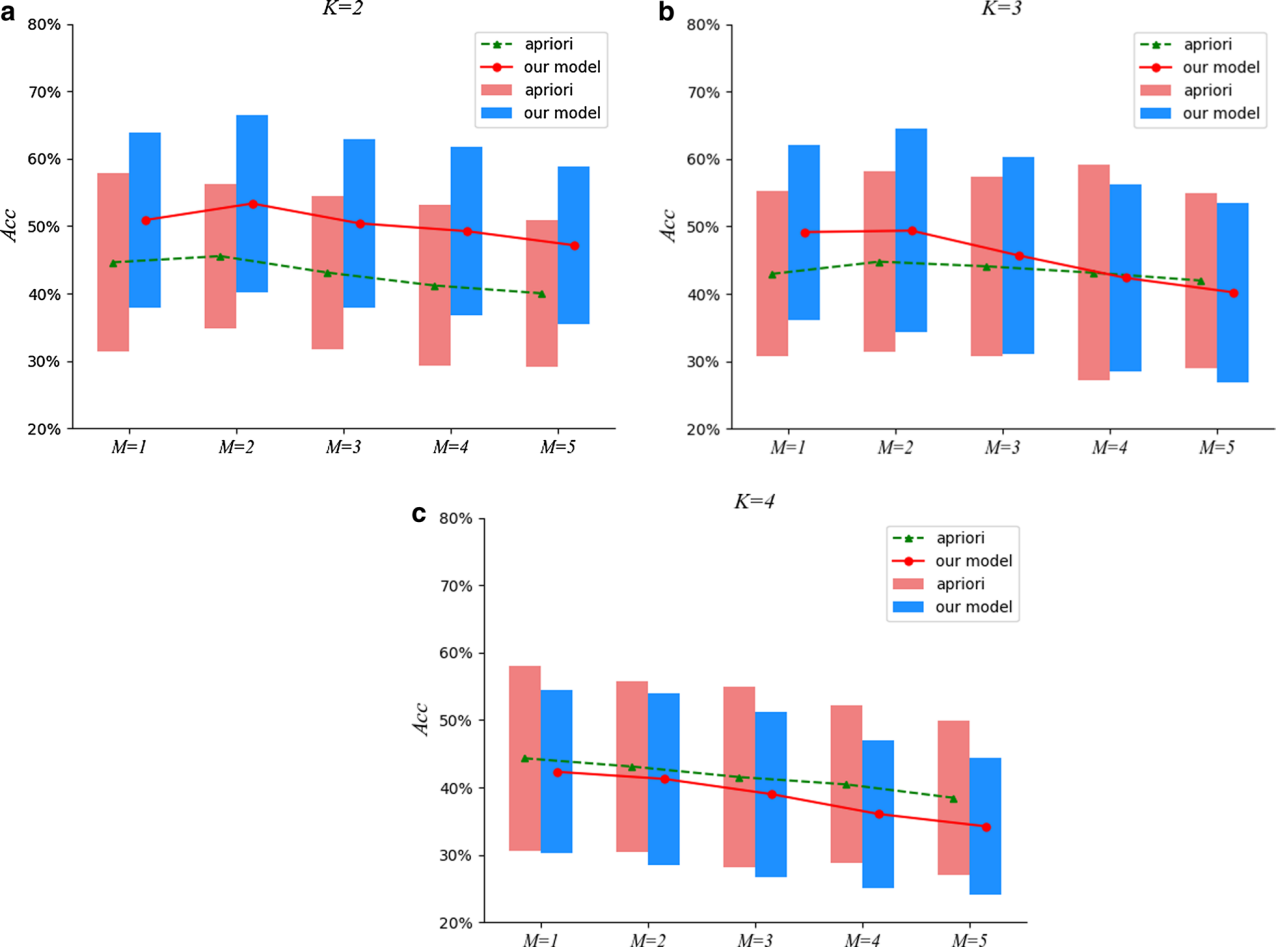
The comparison results under $M=6$

**Fig. 6:**
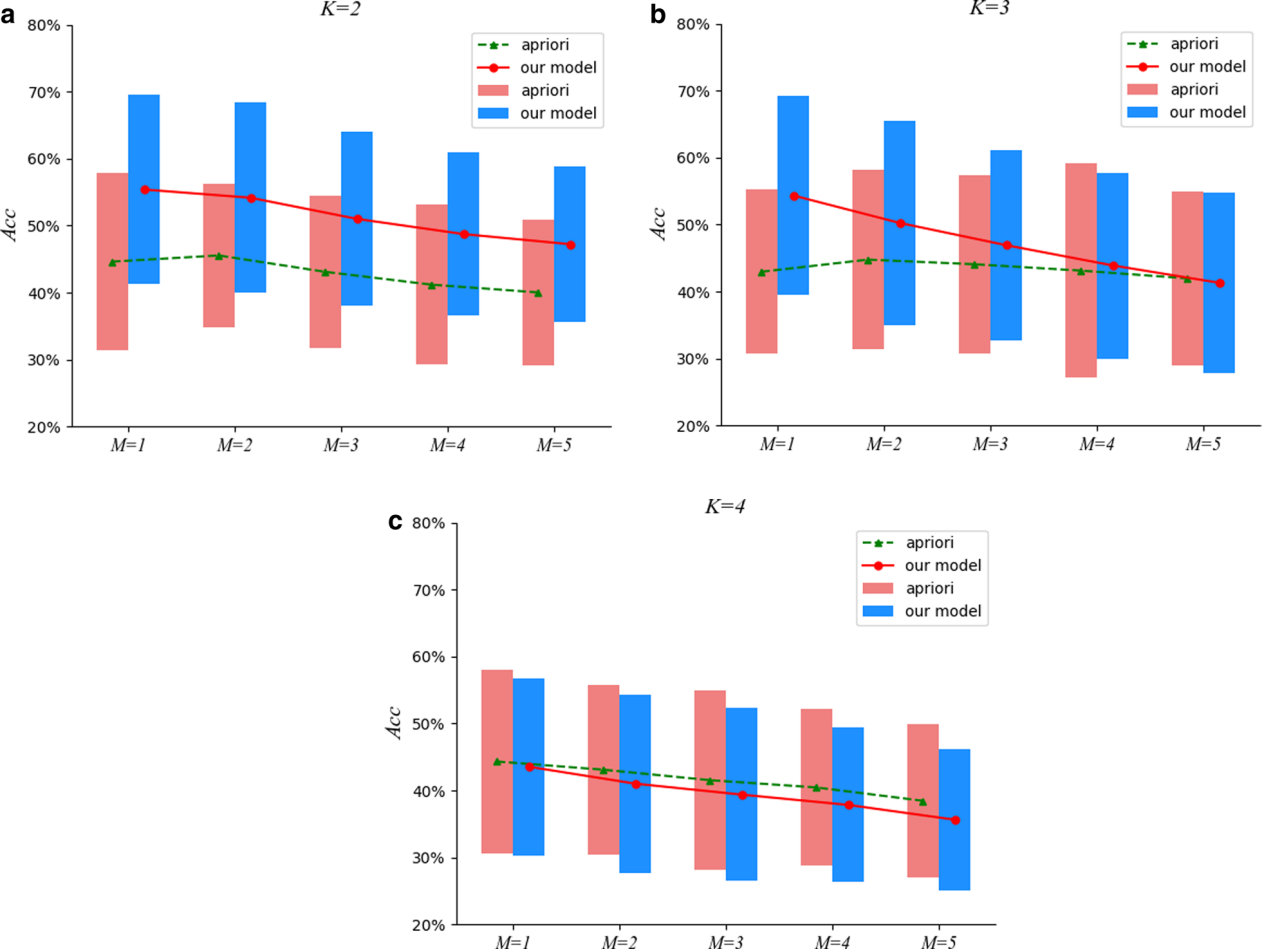
The comparison results under $M=7$

**Fig. 7:**
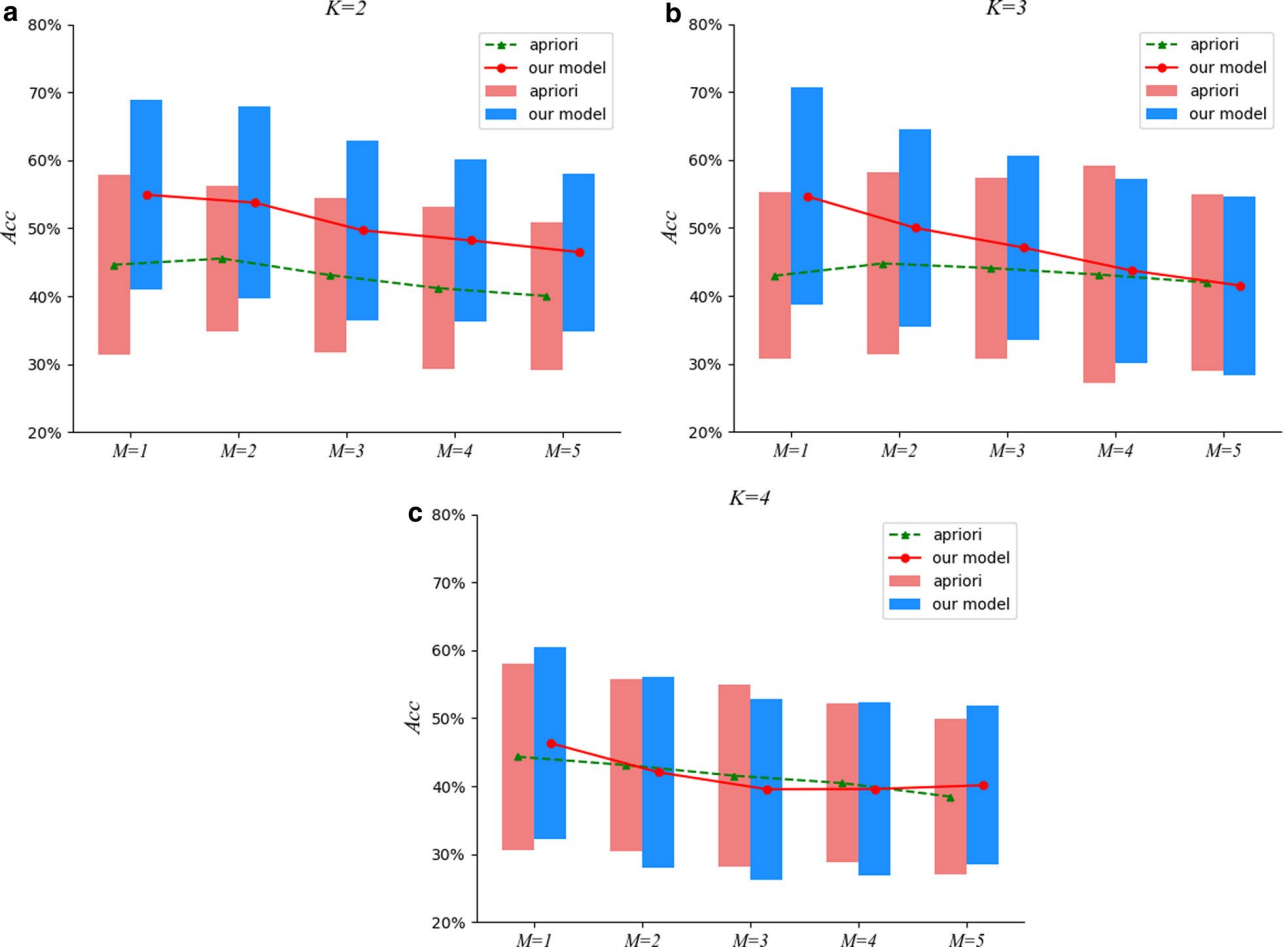
The comparison results under $M=8$

**Fig. 8:**
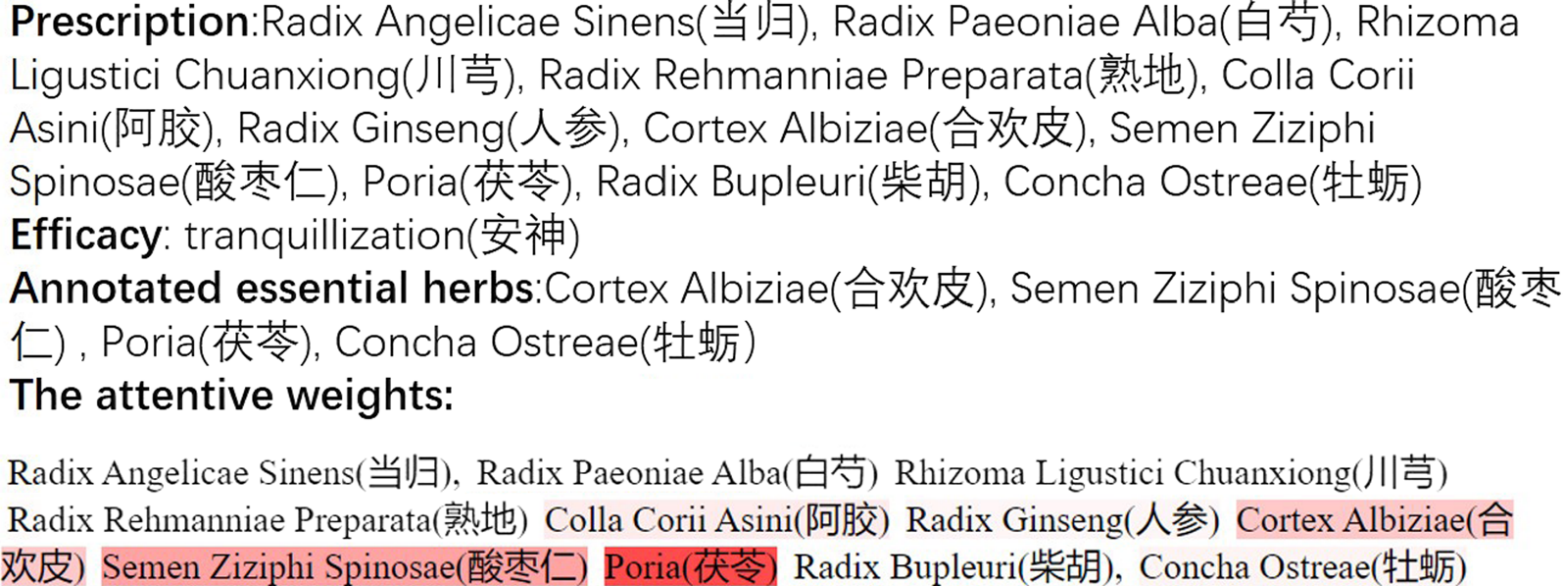
A visualized demonstration of the attentive weights for a sample prescription

**Table 3:** The hyperparameter values

Hyper-parameter name	Value
Epochs	30
batch_size	1
Herb embedding dimension d$^h$	50
Optimization algorithm	Adam
d$^a$	128
Hidden units of full connection layer	50
L2 regular factor	0.0002
Optimization learning rate	0.001

## Methods

As discussed above, our solution to ESHGs detection consists in a two-stage approach, in which the first stage is to capture essential herbs in a prescription for its efficacy thus resulting in a cleaned collection of prescriptions, and the second stage is to discover ESHGs from the distilled prescriptions. The architectural diagram is given in Fig. 1 and will be detailed below in this section.

In order to capture essential herbs in a prescription for its efficacy, we devise a hierarchical attentive neural network with its *sigmoid* output, denoting the probability of a prescription to a given efficacy, and the corresponding attentive weights of the herbs in the prescription expressing their correlations to the efficacy. For a given efficacy, we collect prescriptions with the efficacy as positive samples and prescriptions without the efficacy as negative samples, and train our hierarchical attentive neural network with these samples. Afterwards, the trained model is used to distill the positive samples with the following strategy: the herbs with attentive weights below a prespecified threshold in a positive sample prescription are treated as being unrelated to the efficacy and thus filtered out from the prescription, and the remaining herbs are thought as essential in the prescription for its efficacy. The distilling process in the first stage is then followed by the second stage, as shown in Fig. 1, where association analysis is performed to mine frequent herbal sets in the distilled positive sample collection and the frequent sets are returned as ESHGs of the efficacy.

**Table 4:** The effectiveness of our approach in comparison with the simple Apriori under $N = 8$ and ${min\_sup = 0}$

Efficacy	The simple Apriori			Apriori on distilled prescriptions		
	$K=2$ (%)	$k=3$ (%)	$k=4$ (%)	$k=2$ (%)	$k=3$ (%)	$k=4$ (%)
Activating blood to	10.30	14.09	14.32	15.78±0.18	22.79±0.24	25.77±0.25
Resolve stasis						
Dispersing phlegm	15.45	21.34	23.17	18.08±0.16	25.27±0.18	28.53±0.22
Strengthening bone	8.51	11.83	13.36	11.56±0.14	17.46±0.24	20.42±0.34
Stopping bleeding	18.62	23.58	24.56	19.13±0.27	23.75±0.42	25.27±0.49
Diminishing swelling	5.99	6.77	6.68	6.58±0.15	8.44±0.19	9.42±0.20
Draining dampness	17.98	22.25	22.63	19.48±0.17	23.99±0.20	24.90±0.19
Improving eyesight	10.37	13.67	15.00	13.86±0.18	19.37±0.20	21.52±0.21
Invigorating spleen	17.12	24.46	27.09	21.32±0.17	31.35±0.15	36.21±0.21
Arresting cough	15.81	20.85	22.01	19.78±0.16	27.52±0.26	31.09±0.35
Tranquillization	15.78	20.76	21.97	19.90±0.11	27.72±0.11	31.90±0.13
Relieving pain	8.88	12.27	14.30	9.88±0.20	12.92±0.28	14.30±0.31
Relieving itching	7.74	10.81	11.77	8.39±0.22	11.82±0.38	13.47±0.46
Removing obstruction	14.62	19.68	20.91	18.72±0.07	25.00±0.09	26.93±0.08
in the channels						
The whole average	12.86	17.11	18.29	15.57	21.34	23.83

### Hierarchical attentive neural network model

The hierarchical attentive neural network devised to identify essential herbs in a prescription for its efficacy is shown in Fig. 2. The model is divided into four layers, namely a herb embedding layer, two attention layers and an output layer. We detail these layers separately below.

Let $H\in {\mathbb {R}^{N\times d^h}}$ be the embedding vocabulary with *N* rows corresponding to distinct herbs of TCM, which are expressed in *H *with real-valued vectors of dimensionality $d^h$. For a given prescription $P =\{h_1, h_2, \ldots , h_k\}$ consisting of *k* distinct herbs $h_1$, $h_2$, ..., and $h_k$,where $h_i$ is denoted with the corresponding row index in *H*, the herb embedding layer of our model first extract those row vectors indexed with $h_1$, $h_2$, ..., and $h_k$ from *H* (i.e. the embeddings of those herbs in *H*),and stacks them into a matrix ${\mathcal {H}\in \mathbb {R}^{k\times d^h}}$ as the initial representation of *P* for our model. Afterwards, the matrix ${\mathcal {H}}$ is fed to the two attention layers, where the first attention layer aims to capture correlations among herbs in* P* and enrich every herb’s embedding in ${\mathcal {H}}$ with the correlational information, while the second attention layer attempts to distinguish essential herbs of *P* for its efficacy which are verified by the last layer, i.e. the output layer. The two attention layers together transform the initial representation ${\mathcal {H}}$ of a prescription into a compact vector representation of the prescription to which all the herbs in the prescription contribute in accordance with their final attentive weights (i.e. obtained in the second attention layer), respectively. The last output layer takes the compact representation and outputs a probability of the prescription belonging to its corresponding efficacy. The training algorithm based on gradient descent adapts the parameters in our model to maximize the probability. The attentional weights in the second attention layer for a prescription resulted from the learned model parameters are employed to distill the prescription.

#### The first attention layer

The purpose of the first attention layer is to capture correlations among herbs in a prescription and accordingly update its initial representation in ${\mathcal {H}}$. Concretely, given a prescription *P* and its initial representation ${\mathcal {H}}$, this layer first calculates attentive weights of a herb over all the herbs in *P* to form a weight distribution vector of the dimensionality *k*, and all such vectors of the *k* distinct herbs are collected into the matrix ${\beta \in \mathbb {R}^{k\times k}}$, as shown in formula (1), where $W{_{p1}\in \mathbb {R}^{d^h\times d^h}}$ is the parameter of the model to be learned in the training process. After that, all the initial representation vectors in ${\mathcal {H}}$ are summarized in *P* according to the weight distribution vector of an herb over all the herbs of *P*, resulting in an updated representation for the herb. Formula(2) gives the calculation for all the herbs in a prescription, where ${\bar{\mathcal {H}}\in \mathbb {R}^{k\times d^h}}$ contains the updated representations for all the herbs in *P*.

1 $$\begin{aligned} \beta= & {} softmax(\mathcal {H}W_{p1}\mathcal {H}^T ) \end{aligned}$$

2 $$\begin{aligned} \bar{\mathcal {H}}= & {} \beta \mathcal {H} \end{aligned}$$

#### The second attention layer

The second layer takes the updated representations for all the herbs of *P* in $\bar{\mathcal {H}}$, captures contributions of these herbs for the efficacy of *P* and accordingly formulates feature vector of *P* for fitting its efficacy. The contributions are characterized with the learned weight distribution $\alpha$ in formula (3), where $W{_{p2}\in \mathbb {R}^{d^a\times d^h}}$ and $W{_{p3}\in \mathbb {R}^{1\times d^a}}$ are learnable parameters of the model, and ${\alpha \in \mathbb {R}^k }$ is the weights of all the herbs in *P* for its efficacy. According to these weights, the feature vector $M\in \mathbb {R}^{d^h}$ of * P * for fitting its efficacy is calculated with the weighted sum of all the herbal embeddings in *H*, as shown in formula (4).

3 $$\begin{aligned} \alpha= & {} softmax(W_{p3}\tanh (W_{p2}\bar{\mathcal {H}}^T ) \end{aligned}$$

4 $$\begin{aligned} M= & {} \alpha \mathcal {H} \end{aligned}$$

A prescription, which is treated as a collection of herbs, is mapped to a low-dimensional space by utilizing the weighted sum of all its herbal embeddings, and its feature vector has the same dimensionality as herbal embedding.

**Table 5:** The average precision of each essential-herb-labeled prescription

Prescription	Activating blood to resolve stasis (%)	Invigorating spleen (%)	Arresting cough cough (%)	Tranquillization (%)
1	100.00	100.00	57.42	100.00
2	91.51	95.00	38.54	100.00
3	55.97	74.09	72.09	58.33
4	60.95	75.76	51.44	100.00
5	77.08	94.29	65.12	48.89
6	72.92	100.00	57.78	9.55
7	63.54	83.33	81.00	59.03
$MAP_{e}$	76.95	88.57	64.24	66.86

#### The output layer and the loss function

The final layer, i.e. the output layer, is a typical perceptron, which performs affine transformation on the *M* and the resulted score is squashed into a probability of the prescription belonging to its efficacy. The process is described as in formula (5), where $W{_{p4}\in \mathbb {R}^{d^m\times d^h}}$ and $W{_{p5}\in \mathbb {R}^{d^h\times 1}}$ are learnable parameters of the model, and p$_{e}\in$[0,1] is the probability.

5 $$\begin{aligned} p_{e}=sigmoid(W_{p5}ReLU(W_{p4}M^T ) \end{aligned}$$

The training objective for our model is the standard cross-entropy, shown in formula (6), and the stochastic gradient descent algorithm is employed to achieve the optimization. In formula (6), $y\in \{1,0\}$ is the ground-truth efficacy label of a prescription with 1 denoting the target efficacy for which we are mining ESHGs, and 0 denoting the other efficacies. In addition, we leverage $L_2$ norm of all the model parameters (expressed here with $\omega$) to regularize the cross-entropy loss, and $\lambda$ is the corresponding hyperparameter.

6 $$\begin{aligned} L= & {} -\sum [ylog(p_{e})+(1-y)log(1-p_{e})]\nonumber \\&+\lambda \Vert \omega \Vert _2 \end{aligned}$$

Figure 3 depicts the whole flow of the hierarchical attentive neural network model and different parameters employed therein, including their dimensionalities and the alignments in the dimensionalities.

### ESHGs detection

In order to detect ESHGs for an efficacy, we collect prescriptions with the efficacy as the positive samples and prescriptions without the efficacy as negative samples, and all of them compose our training data set for the efficacy. When convergence emerges in the training of our model, all the herbs in a positive sample with their corresponding attention weight $\alpha _i$ below a prespecified threshold are filtered out and the remaining herbs formulate the corresponding distilled prescription. All the distilled prescriptions for a given efficacy obtained with such manner are fed to the Apropri algorithm for mining frequent herbal sets and such frequent sets are regarded as ESHGs for the given efficacy.

## Results

### Data sets

Empirical evaluation of our method requires two types of data. The first one is the efficacies with their known corresponding ESHGs as the gold standard data, and the second one is the prescriptions accompanying with their efficacies as the data to be mined for detecting ESHGs. Due to the lack of gold standard data widely accepted for our task, we intentionally collect efficacies having known reliable ESHGs from Internet , and invite a TCM expert to evaluate these collected ESHGs, finally obtain a data set of 14 efficacies with their corresponding ESGHs for our evaluation. For instance, the efficacy *“activating blood to resolve stasis*(  )” has a known ESHG “ *(Flos Carthami, Semen Persicae, Rhizoma Ligustici Chuanxiong)”*. For the second type of data, we extract the prescriptions having the efficacies in the gold standard dataset from the prescription database^1^ and thus construct the dataset to be mind to detect ESGHs for the efficacies in the standard dataset.And the numbers and the examples of ESGHs of 14 efficacies are given in Table 1.

For a specific efficacy, we treat prescriptions with the efficacy as positive samples and prescriptions without the efficacy as negative samples. Because of the data skew for positive and negative samples, we utilize an under-sampling method to obtain a balanced dataset in a ratio of positive to negative samples of 1:1 for each efficacy. And the numbers of prescriptions extracted for each of the 14 efficacies are listed in Table 2.

### Evaluation method

The purpose of our work is to capture the essential herbs in a prescription for a given efficacy by a classification process and then discover ESHGs from the essential herbs in prescriptions. Hence, the evaluation metrics are the accuracies of the discovered ESHGs instead of the classification. Due to the lack of gold-standard data widely accepted for our task, we could not find out all possible ESHGs for calculating the *recall* metric.

For a given efficacy, there are multiple gold standard ESHGs, as shown in Table 1, and our algorithm is also likely to generate multiple ESHGs for it according to the support threshold. In order to evaluate the effectiveness of the generated ESHGs, we have to compare them against the corresponding gold standard ESHGs for the same efficacy. Obviously, the evaluation is a comparison between two sets, therefore, we propose to employ Dice coefficient for the evaluation. To be specific, suppose *e* is an efficacy with its gold standard ESHGs $S^e$, $A^e$ is the ESHGs returned by our algorithm for *e*, and the Dice coefficient on *e* is

7 $$\begin{aligned} Dice_e=\frac{2|A^e\bigcap S^e |}{|A^e |+|S^e |} \end{aligned}$$

Because $|S^e|$ is fixed in the evaluation process, we ignore it and the formula (7) becomes

8 $$\begin{aligned} Acc_e=\frac{|A^e\bigcap S^e |}{|A^e | } \end{aligned}$$

The identification accuracy on the whole gold standard test set is

9 $$\begin{aligned} Acc_e=\frac{1}{14}\sum _{e=1}^{14}Acc_e \end{aligned}$$

The above metric is in essence exact-matching-based, i.e. a pair of ESHGs from two sets respectively have to be matched exactly in order to contribute to the accuracy. The exact matching requirement is unreasonable because it treats equally both of the partial matching and mismatching situations, thus it is necessary to incorporate different matching situations into unified accuracy measurement. But unfortunately, the solution is not so obvious due to that we have no any knowledge about alignment between the two sets of ESHGs to be compared. In this paper we employ a greedy strategy with which an ESHG from A$^e$ is aligned with the ESGH from S$^e$ who has maximum overlapping with the ESHG from A$^e$ in their contained herbs. Formally, given an ESHG $h_g$ from A$^e$, its correctness relative to S$^e$ is defined as formula (10).

10 $$\begin{aligned} Correctness(hg)=\max _{hg'\in S^e } \frac{2|hg\bigcap hg'|}{|hg|+|hg'|} \end{aligned}$$

where $|hg \bigcap hg'|$ is the number of the same herbs in *hg* and $hg'$. |*hg*| and $|hg'|$ are the herbal numbers in two ESHGs, respectively.

Based on formula (10), the identification accuracy of our algorithm for a given efficacy *e*, when it identifies $A^e$, but the corresponding gold standard ESHGs is $S^e$, is adapted from formula (8) to formula (11).

11 $$\begin{aligned} Acc_e=\frac{\sum _{hg\in A^e}Correctness(hg)}{|A^e|} \end{aligned}$$

We finally utilize the formula (9) where the $Acc_e$ is calculated through formula (11) to evaluate the effectiveness of our algorithm.

### The hyperparameters

In order to determine the optimal hyperparameters in our model, we leave the efficacy *“heat-clearing and detoxifying*(  )” with its gold standard ESHGs and the corresponding prescriptions out of the data as our development dataset, and based on the development, we set the hyperparameters of our model as shown Table 3. Moreover, the herb embedding vocabulary *H* is initialized randomly, and the attentive weights in 10 runs of the training process are averaged as the final attentive weights of herbs in a prescription to discover essential herbs in the prescription for its efficacy.

### The effect of ESHGs detection

For a given efficacy, its positive and negative samples first pass through the hierarchical attentive neural network and with the model all herbs in these samples obtain their corresponding attentive weights relative to the sample prescription they belong to. After that, all herbs in a positive prescription are ranked in descending order of their attentive weights, and the top *N* herbs are chosen to form the corresponding distilled prescription. In order to improve the quality of the distilled prescriptions, the positive and negative samples are fed into the hierarchical attentive neural network *10* times with its different random initializations of the parameters, thus resulting in *10* groups of the attentive weights. Afterwards, all the *10* attentive weights for an herb in a positive prescription are summed, and the herbs in a positive prescription are ranked in descending order of their summed attentive weights, and the top *N* herbs are retained as the elements of the corresponding distilled prescription.

The resultant distilled prescriptions are then fed to the Apriori algorithm for mining ESHGs of the efficacy. We investigate different settings for the parameter *N*, and also for *K*, the size of an itemset in the Apriori algorithm, and the support threshold *min_sup* in the Apriori algorithm. Furthermore, we also run the Apriori algorithm with the same parameter settings (i.e. the *K* and* min_sup*) on all the positive prescriptions without the distilling process, and compare the results to the corresponding results obtained with the distilling process. Furthermore, in order to verify the stability of our two-stage approach, we perform the two-stage processing *10* times on the same positive and negative samples, and the *10* experimental results in terms of $Acc_e$ for an efficacy are averaged. The average $Acc_e$ and the corresponding *95%* confidence interval are reported hereafter to represent the effectiveness of the approach for an efficacy.

Table 4 gives the average $Acc_e$ of our two-stage approach for the 13 test efficacies, the whole average $Acc_e$ for all these efficacies and the corresponding performances of the simple Apriori algorithm without the distilling process under the conditions $N = 8$ and $min\_sup = 0$ combined with different *K* $\in$ {*2*, *3*, *4*}.

The experimental results in Table 4 shows that our two-stage approach with the distilling process based on the hierarchical attentive neural network consistently outperforms the counterpart approach based on the simple Apriori without the distilling process. Our hierarchical attentive neural network employs two attentive layers to capture the correlations among herbs in a prescription as well as essential herbs in a prescription for its efficacy, which makes the distilled prescription cleaner and clearer in its efficacy description, thus improving the ESHGs detection based on the Apriori algorithm. For instance, for the efficacy *“activating blood to resolve stasis* (  )”, without the distilling process the number of the frequent *2*-itemsets (i.e. $K = 2$) reaches to about *5230*, but the distilling process reduces the number to about *2400* and at the same time the accuracy in the ESHGs detection increases 5.48%. A slight exception among the accuracies of Table 4 arises on the efficacy *“relieving pain*(  )” due to the small-sized positive dataset for this efficacy, which consists of just 71 samples. On the contrary, the efficacies *“dispersing phlegm*(  )” and *“invigorating spleen*(  )”, have much more positive samples, therefore, the resultant ESHGs for them are also much more accurate.

Furthermore, we also investigate empirically the impact of different *min_sup* settings on the effectiveness of ESHGs detection. To be specific, we set *min_sup* to be the *M-th* largest support value among all the *K*-itemsets occurring in the prescription set, and inspect the effectiveness of the Apriori algorithm on the prescription set. Figures 4, 5, 6 and 7 demonstrate comparisons of our two-stage approach with the raw Apriori in the average effects of the 13 efficacies for various combinations of the parameter settings *M*, *K* and *N*.

We can observe from Figs. 4, 5, 6 and 7 that, for $K =2$, our approach performs much better that the raw Apriori algorithm even we adopt aggressive *N* (when *N* is set to be a small value such as * 5*, we aggressively filter the herbs in a prescription and retain at most only *5* herbs in a prescription). However, when $K = 3$ and *4*, the aggressive distilling process leads to an obvious decrease in the performance. As we increase *N *(for instance, $N = 8$ ), the effectiveness is improved even when $K = 3$ and *4*. Therefore, we reported the results in Table 4 with $N = 8$.

### Effect of identifying essential herbs

In this subsection, we further verify the effect of the hierarchical attentive neural network for capturing essential herbs in a prescription for its efficacy. For the purpose we collect some additional prescriptions with efficacies from the 14 ones in Table 1. As the collecting results, we obtain 8 prescriptions for every efficacy from *“activating blood to resolve stasis*(  )”, *“invigorating spleen*(  )”,*“ arresting cough*(  )” and *“tranquillization*(  )”, in total 32 prescriptions. We invite a TCM professional to annotate manually the essential herbs of these prescriptions for their corresponding efficacies. After that, we feed each of them into the trained hierarchical attentive neural network for the corresponding efficacy and therefrom fetch the attentive weights (i.e. the attentive weights of the second attentive layers) for the herbs in a prescription. We sort all the herbs in a prescription in a descending order of their attentive weights, compare them against the annotated essential herbs and accordingly evaluate the effect of our approach for the essential herb detection.

As for the evaluation metric, we employ a traditional one, namely *MAP* (Mean Average Precision), which is widely used for quantitative analysis of ranking algorithms in information retrieval and search engines. Suppose a prescription *p* has $n_p$ essential herbs annotated by the TCM professional, the average precision (*AP*) of the hierarchical attentive neural network on this prescription is defined as:

12 $$\begin{aligned} AP_p=\frac{1}{n}\times \sum _{i=1}^{n_p} \frac{i}{position(i)} \end{aligned}$$

where *position(i)* is the position of the essential herb *i* in the ranking list returned by our hierarchical attentive neural network for the prescription *p*. Furthermore, if an efficacy *e* has $m_e$ prescriptions (here $m_e = 8$ for all the four efficacies), the *MAP* of the neural network for *e* is

13 $$\begin{aligned} MAP_e=\sum _{p=1}^{m_e}AP_p \end{aligned}$$

Table 5 demonstrates the effects of the hierarchical attentive neural network for detecting essential herbs in prescriptions in terms of $AP_p$ and $MAP_e$. In addition, in Fig. 8 we give a visualized demonstration of the attentive weights for a sample prescription with its herbs shaded differently to express their respective attentive weight values. Observing Table 5 and Fig. 8, we can conclude that our hierarchical attentive neural network indeed is able to capture essential herbs in a prescription for its efficacy, gaining more than 60% of the $AP_p$ for majority of the prescriptions. The extreme exception arises for the sixth prescription of the efficacy *“tranquillization*(  )”, for which the $AP_p$ is only 9.55%. In order to get insight into the reason of the extreme exception, we analyze manually the prescription and find that, it is composed of herbs *“Rhizoma Ligustici Chuanxiong*(  ), *Radix Paeoniae Alba*(  ), *Radix Rehmanniae Recens*(  ), *Caulis Spatholobi*(  ), *Folium Mori*(  ), *Flos Chrysanthem*(  ), *Tribulus terrestris*(  ), *Herba Mentha*(  ), *Rhizoma et Radix Notopterygi*(  ), *Semen Ziziphi Spinosae*(  ), *Radix Ginseng* (  )” and labeled wrongly with the auxiliary efficacy *“tranquillization*(  )”, but in fact its primary efficacy should be *“clearing liver heat and restraining liver yang*(  )”. The label noise results in the erroneous essential herbs identified by the hierarchical attentive neural network.

## Discussion

In summary, we utilize a neural network with two attentive layers to identify essential herbs in a prescription for its efficacy, and then discover ESHGs from TCM prescriptions. The efficacy of a prescription is very complicated which is related to herbal combination, herbal dosage, and dosage form. There are still some factors we have not considered. As a successor of this work, we intend to incorporate more herbal information such as dosage into our model to further lift the performance. Furthermore, discovering regularities in TCM prescription composition with data-driven methods and leveraging such regularities to guide the ESHGs detection are another our effort direction. Additionally, integrating the principle of *Jun-Chen-Zuo-Shi* into our model is another interesting point in our further study.

## Conclusion

In this paper we propose a two-stage approach for discovering ESHGs from TCM prescriptions. We devise a neural network with two attentive layers to capture semantical correlations among herbs in a prescription and at the same time identify essential herbs in a prescription for its efficacy. Such attentions are beneficial to overcoming the difficulties when performing data mining on the prescription data accumulated in the long period of TCM history. The detailed experiments verify the effectiveness of our two-stage approach in the whole as well as the hierarchical attentive neural network for identifying the essential herbs in a prescription for its efficacy.

## Data Availability

The datasets used and analyzed during the current study are available from the first author upon reasonable requests.

## Abbreviations

ESHG: Efficacy-specific herbal group
TCM: Traditional Chinese medicine
LDA: Latent dirichlet allocation
MAP: Mean average precision

## Footnotes

1 https://www.zhzyw.com/.
